# Palladium-catalyzed cross coupling reactions of 4-bromo-6*H*-1,2-oxazines

**DOI:** 10.3762/bjoc.5.44

**Published:** 2009-09-16

**Authors:** Reinhold Zimmer, Elmar Schmidt, Michal Andrä, Marcel-Antoine Duhs, Igor Linder, Hans-Ulrich Reissig

**Affiliations:** 1 Freie Universität Berlin, Institut für Chemie und Biochemie, Takustrasse 3, D-14195 Berlin, Germany; 2 Technische Universität Dresden, Institut für Organische Chemie, D-01061 Dresden, Germany

**Keywords:** alkyne, halogenation, 1,2-oxazines, palladium catalysis, pyridines

## Abstract

A number of 4-aryl- and 4-alkynyl-substituted 6*H*-1,2-oxazines **8** and **9** have been prepared in good yields via cross coupling reactions of halogenated precursors **2**, which in turn are easily accessible by bromination of 6*H*-1,2-oxazines **1**. Lewis-acid promoted reaction of 1,2-oxazine **9c** with 1-hexyne provided alkynyl-substituted pyridine derivative **12** thus demonstrating the potential of this approach for the synthesis of pyridines.

## Introduction

A broad range of synthetic applications demonstrates that 1,2-oxazine derivatives constitute a versatile class of N,O heterocycles [1–13]. Considerable attention has been paid to 6*H*-1,2-oxazines **1** bearing a C-4,C-5-double bond [14–18], which are useful intermediates in the synthesis of γ-lactams [19], γ-amino acids [20], amino alcohols [20], aziridines [21], pyrrolizidines [22], and pyrrolidine derivatives [15,23–24]. In the context of our ongoing exploration of the synthetic potential of these heterocycles we were interested to modify the substitution pattern of the C-4,C-5 double bond of 6*H*-1,2-oxazines [25–27]. Herein, we describe our results dealing with the halogenation of 6*H*-1,2-oxazines **1** and the use of the resulting products as precursors in palladium-catalyzed cross coupling reactions.

## Results and Discussion

**Scheme 1:**
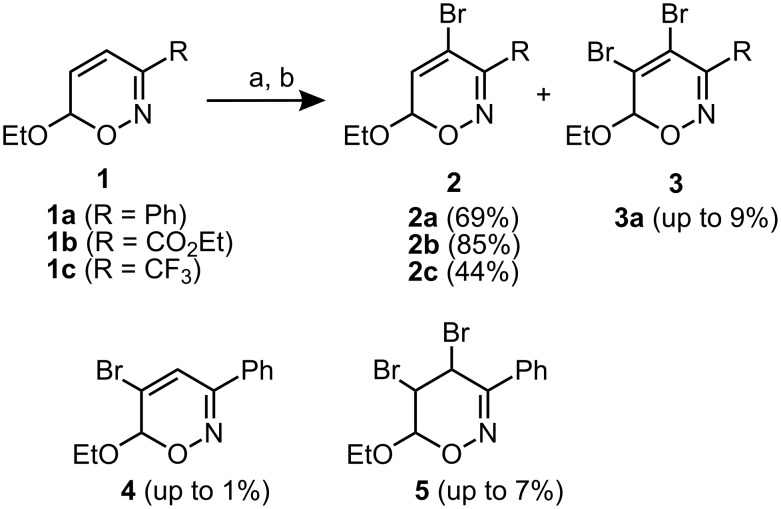
Brominations of 6*H*-1,2-oxazines. a) Br_2_, Et_2_O, −30 °C, 2 h. b) Et_3_N, −30 °C to r.t., overnight.

Not much is known about halogenated 6*H*-1,2-oxazines and only a few mostly inefficient procedures are described [28–32]. This prompted us to investigate a more practical access to halogenated 6*H*-1,2-oxazines. Gratifyingly, the desired 4-bromo-substituted 6*H*-1,2-oxazines **2a**–**2c** could be prepared in a one-pot procedure by bromine addition to precursors **1a**–**1c** [14] and HBr elimination by treatment with triethylamine (Scheme 1). The 4-bromo-6*H*-1,2-oxazines were obtained in reasonable to good yields. The bromination of 3-phenyl-substituted 6*H*-1,2-oxazine **1a** often resulted in a mixture of several brominated products which are easily separable by chromatography. Depending on the reaction scale and the amount of bromine used (1.5 to 3 equiv) by-products such as **3a**, **4** and **5** could be isolated in varying yields. The unexpected formation of 4,5-dibromo-6*H*-1,2-oxazine **3a** can obviously be rationalized by addition of bromine to **2a** and elimination of HBr during the bromination reaction of **1a**.

**Scheme 2:**
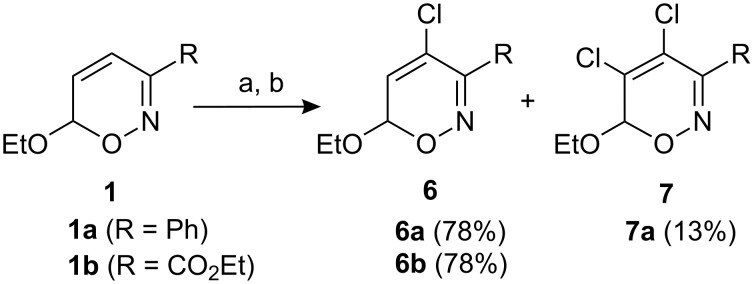
Chlorinations of 6*H*-1,2-oxazines. a) Cl_2_, Et_2_O, −30 °C. b) Et_3_N, −30 °C to r.t.

The literature describes just one related 4-chloro-substituted 6*H*-1,2-oxazine which was prepared by a hetero-Diels–Alder cycloaddition–elimination sequence of 2-chloro-1-nitroso-1-phenyl-ethene and 1-bromo-2-ethoxyethene in low yield (22%) [32]. As demonstrated in Scheme 2, a more efficient approach consists in chlorination of 6*H*-1,2-oxazines **1a**,**b** by addition of chlorine and subsequent base-induced dehydrochlorination. The expected 4-chloro-6*H*-1,2-oxazines **6a**,**b** were obtained in good yields. In analogy to the aforementioned bromination, the chlorination of 3-phenyl-6*H*-1,2-oxazine **1a** also led to dihalogenation furnishing 4,5-dichloro-substituted compound **7a** as a by-product in 13% yield.

**Scheme 3:**
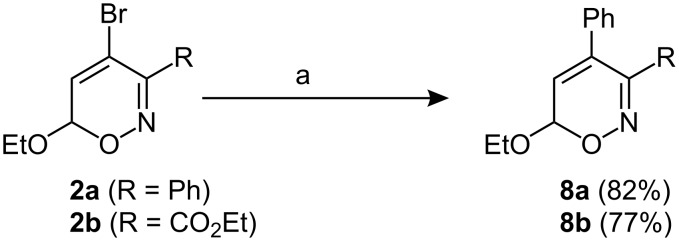
Suzuki-couplings of 4-bromo-6*H*-1,2-oxazines. a) ArB(OH)_2_, Pd(PPh_3_)_4_, Na_2_CO_3_, toluene, 80 °C, 3 h.

With the 4-halogenated 6*H*-1,2-oxazines **2** and **6** in hand, palladium-catalyzed cross couplings offer an efficient and useful approach for the synthesis of novel functionalized 6*H*-1,2-oxazines. The Suzuki-coupling of the 4-bromo-substituted heterocycles **2a**,**b** with phenylboronic acid in the presence of Pd(PPh_3_)_4_ and sodium carbonate at 80 °C in toluene gave the expected 4-phenyl-substituted 6*H*-1,2-oxazines **8a** or **8b** in 82 and 77% yield (Scheme 3).

**Scheme 4:**
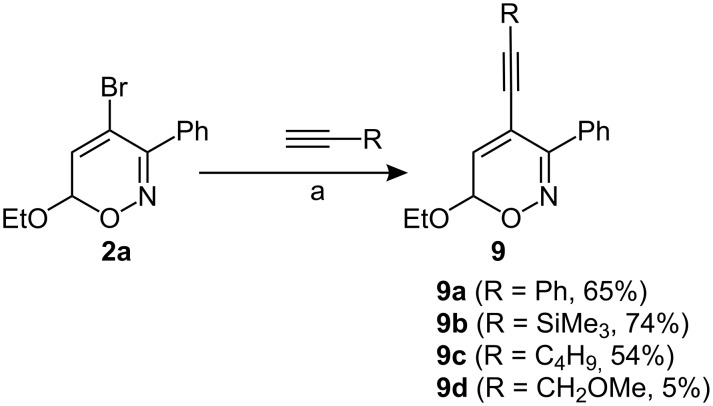
Sonogashira-couplings of 4-bromo-6*H*-1,2-oxazines. a) PdCl_2_(PPh_3_)_2_, CuI, Et_3_N, toluene, r.t., 6–20 h.

4-Bromo-6*H*-1,2-oxazine **2a** also serves as suitable model substrate for Sonogashira-reactions (Scheme 4). When the coupling reaction of **2a** with various terminal alkynes, such as phenylacetylene, trimethylsilylethyne and 1-hexyne, was performed under typical conditions [PdCl_2_(PPh_3_)_2_, CuI, Et_3_N, toluene], the expected 4-alkynyl-substituted heterocycles **9a**–**9c** were isolated in good yields. In contrast, when the same reaction conditions were applied to the coupling of **2a** and methyl propargyl ether, product **9d** was obtained only in very low yield. In addition, Sonogashira coupling of **2a** and methyl propargyl ether performed by an alternative protocol (Pd(OAc)_2_, CuI, PPh_3_, NH*i*Pr_2_ in DMF) afforded the expected product **9d** and a byproduct bearing a 4-enyne moiety at 4-position. This indicates an addition of a second alkyne molecule to the primary product **9**. Similar results were observed for the Sonogashira reaction of **2a** with propargylic alcohol [33].

**Scheme 5:**
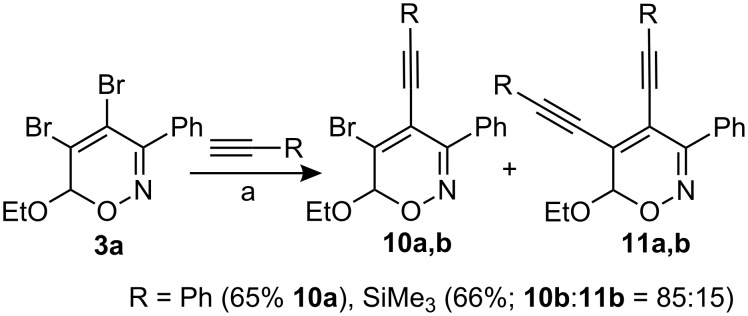
Sonogashira-couplings of 4,5-dibromo-6*H*-1,2-oxazines. a) PdCl_2_(PPh_3_)_2_, CuI, Et_3_N, toluene, r.t., 4 h to overnight.

After successful simple cross couplings of mono-halogenated **2**, the 4,5-dibromo-3-phenyl-6*H*-1,2-oxazine **3a** seemed to be an attractive candidate for a twofold Sonogashira reaction (Scheme 5). Treatment of **3a** with an excess of phenylacetylene under conditions as described in Scheme 4 provided 5-bromo-4-alkynyl-substituted 6*H*-1,2-oxazine **10a** as single product in 65% yield. When the Sonogashira coupling was performed with trimethylsilylethyne under the same reaction conditions an inseparable 85:15-mixture of mono-alkynylated product **10b** and bis-alkynylated compound **11b** was obtained in reasonable yield. These reactions certainly deserve further optimization, however, they already show the potential of compounds such as **3a** to serve as precursors for two subsequent coupling reactions.

## Conclusion and Perspective

**Scheme 6:**
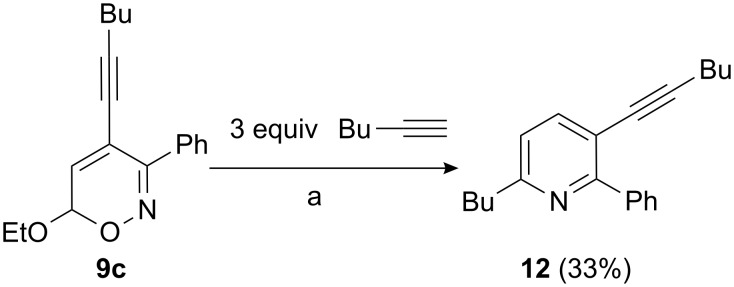
Preparation of trisubstituted pyridine derivatives: a) BF_3_·OEt_2_, CH_2_Cl_2_, −78 °C to r.t., overnight.

In conclusion, we have successfully demonstrated that a series of 4-aryl- and 4-alkynyl-substituted 6*H*-1,2-oxazines **8**, **9**, and **10** are easily accessible in short reaction sequences starting from precursors **1**. These 6*H*-1,2-oxazines should allow access to many interesting five- and six-membered heterocycles. As illustrated in Scheme 6, the 4-hex-1-ynyl-3-phenyl-6*H*-1,2-oxazine **9c** can be converted into the trisubstituted pyridine derivative **12** by treatment of **9c** with boron trifluoride etherate in the presence of an excess of 1-hexyne via an azapyrylium intermediate [34–35]. Additional investigations are required to optimize the preparation diynes of type **11**. Conversion of the new functionalized 6*H*-1,2-oxazines to highly substituted pyridine derivatives will also be reported in due course.

## Experimental

### Bromination of 6*H*-1,2-oxazine **1a**, typical procedure

6*H*-1,2-Oxazine **1a** (5.35 g, 26.3 mmol) was dissolved in diethyl ether (200 mL) and treated with bromine (2.75 mL, 53.7 mmol) at −30 °C under argon atmosphere. After 2 h Et_3_N (54.0 mL, 390 mmol) was added. The reaction mixture was warmed to r.t. overnight and quenched with water (100 mL). The aqueous phase was extracted with CH_2_Cl_2_ (2 × 50 mL) and the combined organic phases were dried with Na_2_SO_4_. Purification of the crude product by column chromatography (SiO_2_, hexane:EtOAc 8:1, then 4:1) gave the 4-bromo-substituted 6*H*-1,2-oxazine **2a** (5.11 g, 69%), the 4,5-dibromo-substituted by-product **3a** (0.821 g, 9%), and starting material **1a** (0.335 g, 6%).

4-Bromo-6-ethoxy-3-phenyl-6*H*-1,2-oxazine (**2a**): yellow–brown oil. ^1^H NMR (CDCl_3_, 300 MHz): δ = 1.23 (t, *J* = 7.1 Hz, 3 H, CH_3_), AB part of ABX_3_ system (δ_A_ = 3.68, δ_B_ = 3.95, *J*_AX_ = *J*_BX_ = 7.1 Hz, *J*_AB_ = 9.8 Hz, 2 H, OCH_2_), 5.58 (d, *J* = 5.2 Hz, 1 H, 6-H), 6.70 (d, *J* = 5.2 Hz, 1 H, 5-H), 7.35–7.50, 7.50–7.60 (2 m, 3 H, 2 H, Ph) ppm. ^13^C NMR (CDCl_3_, 75.5 MHz): δ = 14.8 (q, CH_3_), 64.3 (t, OCH_2_), 94.7 (d, C-6), 112.9 (s, C-4), 127.8, 128.0, 128.8, 129.7, 133.1 (4 d, s, Ph, C-5), 156.2 (s, C-3) ppm. For the complete characterization, see ref. [31].

4,5-Dibromo-6-ethoxy-3-phenyl-6*H*-1,2-oxazine (**3a**): brown oil. ^1^H NMR (CDCl_3_, 300 MHz): δ = 1.25 (t, *J* = 7.1 Hz, 3 H, CH_3_), AB part of ABX_3_ system (δ_A_ = 3.76, δ_B_ = 3.97, *J*_AX_ = *J*_BX_ = 7.1 Hz, *J*_AB_ = 9.7 Hz, 2 H, OCH_2_), 5.71 (s, 1 H, 6-H), 7.38–7.48, 7.49–7.56 (2 m, 3 H, 2 H, Ph) ppm. ^13^C NMR (CDCl_3_, 75.5 MHz): δ = 14.7 (q, CH_3_), 65.0 (t, OCH_2_), 99.8 (d, C-6), 114.4, 124.8 (2 s, C-4, C-5), 128.1, 128.9, 129.9, 133.3 (3 d, s, Ph), 155.9 (s, C-3) ppm. IR (neat): 3065–2900 (=C–H, C–H), 1630 (C=N), 1600 (C=C) cm^−1^. HRMS (80 eV, 40 °C) *m*/*z* calcd for C_12_H_11_^79^Br_2_NO_2_: 358.9157; found: 358.9160.

### Chlorination of 6*H*-1,2-oxazine **1b**, typical procedure

Chlorine gas was passed into diethyl ether (28 mL) at −30 °C until the solution became dark yellow. Then, 6*H*-1,2-oxazine **1b** (0.200 g, 1.00 mmol) was added and the reaction mixture was monitored by TLC; upon complete consumption, triethylamine (2.00 mL, 27.8 mmol) was added at −30 °C and the mixture was slowly warmed to r.t. After addition of brine, the phases were separated, the aqueous phase was extracted with CH_2_Cl_2_ (2 × 20 mL) and the combined organic phases were dried with Na_2_SO_4_. Column chromatography (SiO_2_, hexane, hexane:EtOAc 9:1, then 4:1) afforded the 4-chloro-substituted product **6b** (0.182 g, 78%) as pale–yellow oil.

Ethyl 4-chloro-6-ethoxy-6*H*-1,2-oxazine-3-carboxylate (**6b**): ^1^H NMR (CDCl_3_, 300 MHz): δ = 1.22 (t, *J* = 7.1 Hz, 3 H, CH_3_), 1.40 (t, *J* = 7.2 Hz, 3 H, CH_3_), AB part of ABX_3_ system (δ_A_ = 3.68, δ_B_ = 3.95, *J*_AX_ = *J*_BX_ = 7.1 Hz, *J*_AB_ = 9.6 Hz, 2 H, OCH_2_), 4.40 (q, *J* = 7.2 Hz, 2 H, OCH_2_), 5.72 (d, *J* = 5.0 Hz, 1 H, 6-H), 6.34 (d, *J* = 5.0 Hz, 1 H, 5-H) ppm. ^13^C NMR (CDCl_3_, 75.5 MHz): δ = 13.9, 14.7 (2 q, CH_3_), 62.5, 64.6 (2 t, OCH_2_), 95.3 (d, C-6), 121.1 (s, C-4), 122.7 (d, C-5), 148.5 (s, C-3), 160.3 (s, C=O) ppm. IR (neat): 3105–2975 (=C–H, C–H), 1745 (C=O), 1615 (C=N) cm^−1^. C_9_H_12_ClNO_4_ (233.7): calcd. C, 46.27; H, 5.18; N, 5.99; found: C, 46.35; H, 5.16; N, 6.08.

### Suzuki-coupling of 4-bromo-substituted 6*H*-1,2-oxazine **2a**, typical procedure

6*H*-1,2-Oxazine **2a** (0.0935 g, 0.33 mmol), phenylboronic acid (0.122 g, 1.00 mmol) and Pd(PPh_3_)_4_ (0.016 g, 0.0138 mmol) were dissolved in a mixture of toluene/MeOH (3 mL/0.75 mL) in a heat-gun-dried and argon-flushed flask. A 2M Na_2_CO_3_ solution (1.5 mL) was finally added and the reaction mixture was heated for 15 h at 80 °C. Then, the reaction mixture was cooled to r.t. and washed with 2M Na_2_CO_3_ (with 1% NH_3_) solution. After separation of the phases, the aqueous phase was extracted with CH_2_Cl_2_ (3 × 5 mL) and the combined organic phases were dried with Na_2_SO_4_. The crude product was purified by column chromatography (SiO_2_, hexane:EtOAc 9:1, then 4:1) to afford the Suzuki product **8a** (0.076 g, 82%) as a pale–yellow solid, mp 68–70 °C.

6-Ethoxy-3,4-diphenyl-6*H*-1,2-oxazine (**8a**): ^1^H NMR (CDCl_3_, 300 MHz): δ = 1.25 (t, *J* = 7.1 Hz, 3 H, CH_3_), AB part of ABX_3_ system (δ_A_ = 3.75, δ_B_ = 4.01, *J*_AX_ = *J*_BX_ = 7.1 Hz, *J*_AB_ = 9.8 Hz, 2 H, OCH_2_), 5.73 (d, *J* = 4.9 Hz, 1 H, 6-H), 6.37 (d, *J* = 4.9 Hz, 1 H, 5-H), 7.05–7.10, 7.15–7.27, 7.30–7.35 (3 m, 4 H, 4 H, 2 H, Ph) ppm. ^13^C NMR (CDCl_3_, 75.5 MHz): δ = 15.0 (q, CH_3_), 64.2 (t, OCH_2_), 93.0 (d, C-6), 124.0 (d, C-5), 128.0, 128.2, 128.4, 128.7, 129.0, 130.2, 133.9, 136.5 (5 d, 3 s, Ph, C-4), 157.7 (s, C-3) ppm. IR (KBr): 3040–2930 (=C–H, C–H), 1620 (C=N), 1600 (C=C) cm^−1^. C_18_H_17_NO_2_ (279.3): calcd. C, 77.39; H, 6.13; N, 5.01; found: C, 77.82; H, 6.37; N, 5.08.

### Sonogashira-coupling of 4-bromo-substituted 6*H*-1,2-oxazine **2a**, typical procedure

6*H*-1,2-Oxazine **2a** (0.850 g, 3.19 mmol), trimethylsilylethyne (0.87 mL, 6.17 mmol), PdCl_2_(PPh_3_)_2_ (0.114 g, 0.16 mmol), CuI (0.019 g, 0.10 mmol) and Et_3_N (1.3 mL) were dissolved in toluene (15 mL) in a heat-gun-dried and argon-flushed flask and the reaction mixture was stirred at r.t. for 20 h. The reaction mixture was quenched with water (5 mL). The aqueous phase was extracted with CH_2_Cl_2_ (3 × 10 mL) and the combined organic phases were dried with Na_2_SO_4_. Purification of the crude product by column chromatography (SiO_2_, hexane: EtOAc 20:1, then 4:1) afforded the 4-alkynyl-substituted 6*H*-1,2-oxazine **9b** (0.711 g, 74%) as a colorless oil.

6-Ethoxy-3-phenyl-4-(trimethylsilylethynyl)-6*H*-1,2-oxazine (**9b**): ^1^H NMR (CDCl_3_, 250 MHz): δ = 0.71 (s, 9 H, SiMe_3_), 1.21 (t, *J* = 7.1 Hz, 3 H, CH_3_), AB part of ABX_3_ system (δ_A_ = 3.68, δ_B_ = 3.96, *J*_AX_ = *J*_BX_ = 7.1 Hz, *J*_AB_ = 9.7 Hz, 2 H, OCH_2_), 5.62 (d, *J* = 5.1 Hz, 1 H, 6-H), 5.69 (d, *J* = 5.1 Hz, 1 H, 5-H), 7.34–7.44, 7.67–7.73 (2 m, 3 H, 2 H, Ph) ppm. ^13^C NMR (CDCl_3_, 125.8 MHz): δ = −0.7 (q, SiMe_3_), 14.9 (q, CH_3_), 64.2 (t, OCH_2_), 92.0 (d, C-6), 99.5, 101.9 (2 s, C≡C), 114.0 (s, C-4), 127.7, 128.7, 129.5, 130.1, 132.9 (4 d, s, Ph, C-5), 155.5 (s, C-3) ppm. IR (neat): 3085–2900 (=C–H, C–H), 2160 (C≡C), 1620 (C=C), 1580 (C=N) cm^−1^. C_17_H_21_NO_2_Si (299.5): calcd. C, 68.19; H, 7.07; N, 4.68; found: C, 68.17; H, 7.08; N, 4.74.
